# Error performance analysis of generalized quadrature spatial modulation using H-8QAM

**DOI:** 10.1038/s41598-022-24950-8

**Published:** 2022-11-30

**Authors:** Nathael Sibanda, Hongjun Xu, Narushan Pillay

**Affiliations:** School of Engineering, University of KwaZulu Natal, Durban, 4001 South Africa

**Keywords:** Electrical and electronic engineering, Aerospace engineering, Mechanical engineering

## Abstract

Motivated to enhance the error performance (*EP*) of generalised complex quadrature spatial modulation (GCQSM) systems, this study proposes a scheme that builds on GCQSM and uses hexagonal quadrature amplitude modulation (H-QAM) constellations which have the advantages of a maximised Euclidean distance with relatively low peak-to-average power ratio, compared to conventional QAM (C-QAM) systems. This in turn, leads to an enhancement of the *EP* of GCQSM schemes. The proposed scheme utilises a rotated hexagonal 8QAM (H-8QAM) set. Thus, the proposed scheme is herein named; Generalised QSM using H-8QAM (GQSM-H-8QAM). In this study, the *EP* of the proposed GQSM-H-8QAM scheme is investigated over Rayleigh frequency flat-fading channels with additive white Gaussian noise. Additionally, a theoretical average bit error probability (*ABEP*) expression of the GQSM-H-8QAM scheme is formulated and validated using Monte Carlo simulations. Compared to simulation results, the *ABEP* proves to be increasingly tight at high signal-to-noise ratio values. Obtained simulation results also show an improvement in the *EP* of the GQSM-H-8QAM scheme over various SM schemes like GCQSM, C-QSM and conventional-generalized spatial modulation (C-GSM), at the same spectral efficiency (*SE*). An improvement in the *EP* of 0.61 dB with *SE* of 8 bits/s/Hz is seen in $4\times 4$ GQSM-H-8QAM over $4\times 4$ GCQSM using C-8QAM, 2.58 dB over $4\times 4$ C-QSM-C-64QAM and a gain of 4.85 dB over $4\times 4$ C-GSM-C-64QAM.

## Introduction

Nowadays, the world has been forced to adapt to the the Covid-19 living conditions. Thus, there has been a high demand and compulsory need for the human race to work at home/indoors with no/minimum physical interaction. This has resulted in a high dependence on the internet and wireless communications networks. Hence, the need for wireless communications with reliable link margins and high data rates. Multiple-input multiple-output (MIMO) systems have been shown to improve/enhance spectral efficiency (*SE*) and /or improve the link reliability of wireless networks against multi-path fading^1^. The idea behind MIMO systems is to improve the error performance (*EP*) of wireless communication networks using spatial diversity and/or enhancing *SE* using spatial multiplexing^1^.

Conventional spatial modulation (C-SM) is one of many MIMO techniques that can improve *SE* by the utilization of both the spatial constellation (transmit antenna indices) and the signal constellation^2^. In C-SM, information bits are categorized into two; the bits for transmitting antenna indices and the symbols bits^2^. Only a single transmit antenna per channel use is needed in C-SM. Hence, compared to other transmission schemes like Alamouti space-time block codes and vertical Bell layered space-time scheme (V-BLAST), C-SM was found to have a better *EP* because it precludes inter-channel interference *ICI* and inter-antenna synchronization^3^. Also, C-SM was found to be more bandwidth efficient when compared to conventional modulation with a single transmit antenna^4^. This is because it’s specified transmit antenna per time slot, also conveys additional information^4^. However, the number of physical antennas that can be employed at the transmission side limits the *SE* of C-SM schemes, and thus, conventional generalized spatial modulation (C-GSM) was proposed to alleviate this disadvantage^5^. C-GSM overcomes the C-SM disadvantage by curbing the restriction of transmit antennas being a power of two in C-SM schemes^5^. Thus, in C-GSM, the input data stream bits are mapped into two categories, the symbol signal bits and the spatial constellation bits (antenna pairs combination bits). In C-GSM, a unique combination/grouping of active transmit antennas per time slot is represented by an index. This unique combination/grouping relies on the input random data stream^5^. Thus, C-GSM is different from C-SM as two or more transmit antennas are activated per channel use as compared to one active transmit antenna per channel use in C-SM^5^. Hence, C-GSM enhances the overall *SE* by $\log _2(N_c)$; where $$N_c=\lfloor \log _{2}\begin{pmatrix} N_T \\ N_A\end{pmatrix} \rfloor _{2^p}$$ pairs of permissible transmit antennas for transmission, with $\lfloor \cdot \rfloor _{2^p}$ representing the largest integer that is less than or equal to $(\cdot )$, (that is, an integer *p* power of 2). Whereas, $$\begin{pmatrix} \cdot \\ \cdot \end{pmatrix}$$ represents the binomial coefficient of the argument. $N_T$ is the total number of transmit antennas for the C-GSM scheme, and $N_A$ has been discussed before in^5^. The chosen value of use for $N_A$ in this paper, is 2.

In the past decade, an effort to improve the *EP* and/or *SE* of SM systems, led to the derivation of conventional-quadrature spatial modulation (C-QSM) in Mesleh et al.^6^. C-QSM extends the spatial constellation into two dimensions (the in-phase (*I*) and quadrature (*Q*) dimensions). The *I* dimension is for transmitting the real part of a single amplitude/phase modulated symbol, and the *Q* dimension is for transmitting the imaginary part of the single amplitude/phase modulated symbol^6,7^. The *I* components are modulated into cosine carriers and *Q* components are modulated into sine carriers, respectively. Hence with that, C-QSM eliminates *ICI*, thereby enhancing the *EP* of SM systems^8^. Additionally, C-QSM enhances the *SE* ($\mathfrak {m}$) by $\log _2(N_T)$ *bits*/*s*/*Hz* compared to the C-SM, which achieves $\mathfrak {m}=\log _2(M) + \log _2(N_T)$ *bits*/*s*/*Hz*; where *M* is the amplitude/phase modulation order^6^. In Li et al.^9^, C-QSM is used where indexes of the designated receive antennas ($N_R$) are used to convey information. Thus, in Li et al.^9^, an amplitude/phase modulated symbol is precoded so that only a single receive antenna is activated, thereby conveying more information to the receiver. Also, in Kim^10^, C-QSM has been used in antenna selection schemes to improve the *EP* and simultaneously reduce the detection complexity. However, despite the capability of improving the *EP* and *SE* of SM wireless communication networks, C-QSM has a disadvantage of utilising many transmit antennas as compared to conventional spatial multiplexing (C-SMux) techniques, thereby governing the improvement of the *EP* of SM systems^11^.

### Motivation

Motivated to improve the *EP* and/or *SE* of SM systems with a minimal number of transmit antennas, a generalized complex quadrature spatial modulation (GCQSM) scheme was proposed in Mohaisen et al.^12^. The GCQSM scheme builds on C-QSM by adding the attributes of C-GSM to the C-QSM scheme. It improves the *SE* by transmitting two amplitude/phase modulated symbols drawn from two different constellation sets at each channel use, using unique combinations of generalized *I* domain antennas and *Q* domain antennas. The two symbols could be transmitted from the same antenna at one point, and thus the total modulation set at the transmitter becomes the Minkowski sum of the original two constellation sets^12^. Therefore, compared to C-SM and C-QSM, this leads to an increase in the size of the modulation set and a decrease in the minimum distance between transmitted symbols at the transmitter of GCQSM systems, which in turn results in the degradation of the *EP* of the GCQSM scheme^13^. This results in a poor *EP* because the detector depends on the minimum Euclidean (*MED*) distance between the transmitted vector symbols^14^.

To further enhance/improve the *EP* of SM systems, we propose to build on GCQSM by equipping it with hexagonal QAM (H-QAM) constellations which have the benefits of a maximised *MED* (*M-MED*) with a relatively low peak-to-average (*PA*) power ratio^15^. H-QAM systems are QAM systems with a hexagonal structure and they are densely packed with a *M-MED*. In^15^, it was found that the structure of H-QAM systems gave them an advantage of outperforming conventional-QAM (C-QAM) systems by approximately 0.6 dB. SM and QSM systems with H-QAM were introduced by Cogen et al. in^16^ and in^17^. H-QAM was found to be more energy efficient than square QAM, rectangular QAM and cross-QAM. Hence, its use was found for many applications including multi-carrier systems, MIMO systems, SM, QSM and advanced channel coding^15^ and^17^. Cogen et al.^17^, also solved the error floor degradation of the *EP* of QSM systems by rotating the H-QAM symbols. Also, in^12^, Mohaisen et al. investigated the rotation of symbols in GCQSM systems which also led to an improvement in the *EP* of GCQSM systems. In Naidoo et al.^14^, and Singya et al.^15^, the *EP* of SM systems was also found to depend on the *M-MED* between two neighbouring constellation points and the average symbol energy. Hence, we propose to use H-QAM systems as they have been proven to have a *M-MED* and relatively low *PA* which improves the *EP* of SM schemes, as compared to C-QAM systems.

Thus, to improve the *EP* of SM systems, we propose a GQSM scheme that builds on GCQSM^13^, by combining it with rotated H-QAM systems like Cogen et al.^17^. The proposed scheme adopts rotated hexagonal-8QAM (H-8QAM) and utilises generalized combinations of the *I* and *Q* domain antennas. This is because H-8QAM has a *M-MED* between neighbouring symbols and a relatively low *PA* as compared to C-8QAM^18^. Also, the proposed scheme uses generalized antenna combinations because they help enhance and/or improve *EP* and *SE*^5^. Thus, the benefits of GCQSM and rotated H-QAM systems lead to an improved *EP*. The H-8QAM concept could be extended to other various H-QAM constellations like H-32QAM and others, however as discussed in^18^, this results in a poor *EP* as the size of the hexagonal lattice structure increases. Hence the proposed scheme utilises only H-8QAM as it is the optimum H-8QAM compared to others of the same family^18^. A constellation comparison diagram between rotated H-8QAM and C-8QAM is included in "Appendix 1" of this paper. The creation of H-8QAM symbols was discussed in^18^. It was also well covered including the solution of the error floor in the *EP* of QSM systems by Cogen et al.^17^. This paper adopts the same way of creation and rotation of H-8QAM symbols like in^17^.

In summary, this paper, we propose a GQSM scheme that improves the *EP* of SM systems by equipping GCQSM systems with rotated H-8QAM^17^. The proposed scheme uses the C-QSM approach of splitting two rotated H-8QAM symbols into their respective *I* and *Q* components. The split symbols are then transmitted by generalized combinations of the *I* and *Q* domain antennas like in C-QSM. This helps with eliminating *ICI* and thereby further enhancing the *EP* of the proposed scheme, since the *I* and *Q* components of the C-QSM scheme are modulated into cosine and sine carriers^8^. Thus, the main core contributions of this paper are:

- Proposal of a new GQSM scheme called GSM with H-QAM (GQSM-H-8QAM), that improves the *EP* of GCQSM systems by equipping them with rotated H-8QAM. The H-8QAM systems have a lattice structure that has a *M-MED* and relatively low *PA* compared to C-QAM. This helps improve the *EP* of SM schemes.
- Derivation of an upper bound average bit error rate (ABER) expression for the GQSM-H-8QAM scheme over independent and identically distributed (i.i.d) Rayleigh frequency-flat *RF-F* fading channels.
- Validation of the derived analytical bound using Monte Carlo simulation results.

**Figure 1:**
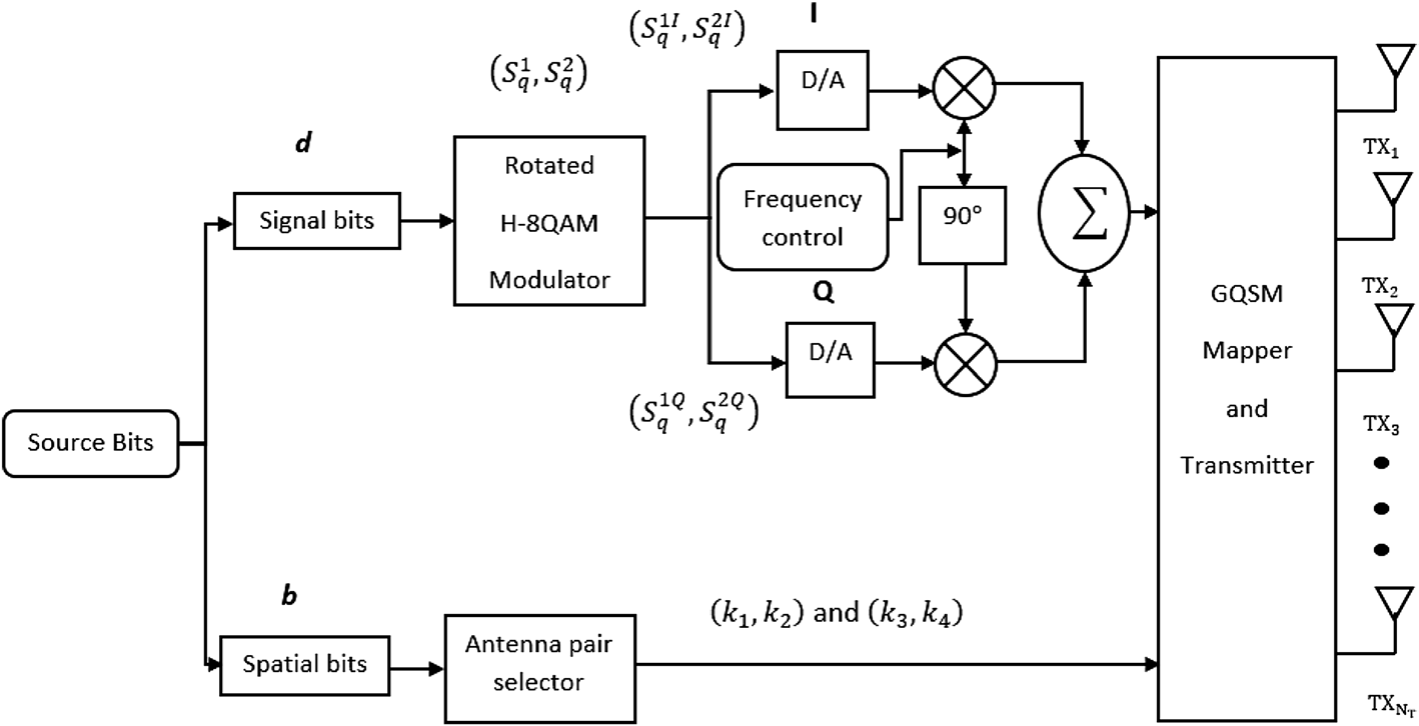
Transmission side of the proposed GQSM-H-8QAM system model.

**Figure 2:**
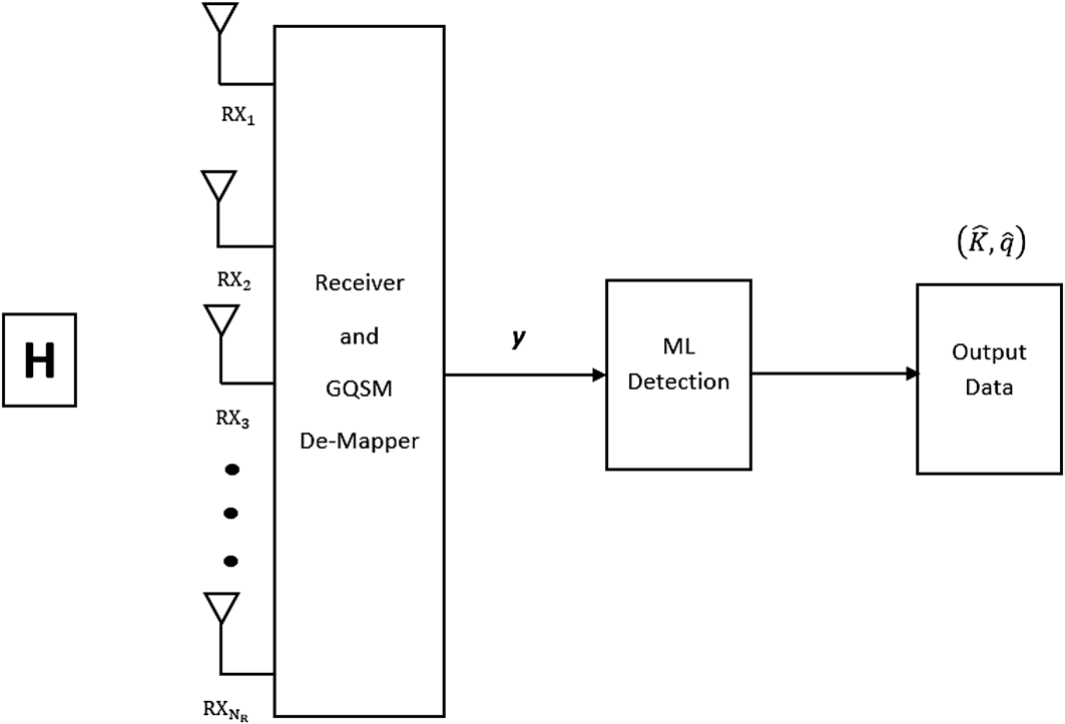
Receiver side of the proposed GQSM-H-8QAM system model.

Finally, the paper is organised as follows. We introduce our proposed GQSM-H-8QAM system in Section "System model". It is presented in the form of a MIMO $N_T \times N_R$ H-8QAM system shown in Figs. 1 and 2. For this system, there exists $$\mathfrak {n}=\begin{pmatrix}N_T \\ N_A\end{pmatrix}$$ possible transmission antenna pairs of which only $N_c$ pairs are allowed for transmission, with $N_c\le \mathfrak {n}$. The receiver computational complexity analysis (CCA) based on real valued multiplications and additions of the GQSM-H-8QAM scheme is then presented in Section "MLD CCA of the proposed GQSM-H-8QAM scheme-based on real valued multiplications and additions", followed by the performance analysis of the proposed scheme in Section "Proposed error performance analysis of GQSM-H-8QAM". Section "Simulation and numerical results analysis" then dwells on the results and performance comparisons. Finally, we conclude the paper in Section "Conclusion".

### **Notation**

$(\cdot )^*$ represents the conjugate and $|\cdot |$ denotes the Euclidean norm operator. We use bold lowercase letters to denote column vectors and uppercase letters for matrices, respectively. Scalar quantities are then represented by regular letters. For a set of $S\times L$ complex matrices, we use $\mathbb {C}^{S\times L}$. For complex arguments, we use $\Re (\cdot )$ and $\Im (\cdot )$ to represent the real and imaginary parts, respectively. $Q(\cdot )$ is for representing the Gaussian Q-function, $E\{\cdot \}$ is for denoting the expectation operator, $\underset{w}{{\text {argmin}}}\{{\cdot }\}$ and $\underset{w}{{\text {argmax}}}\{{\cdot }\}$ represent the minimum or maximum value of an argument with respect to *w*, respectively. $[\cdot ]^T$ and $(\cdot )^H$ are the transpose and Hermitian operators, respectively. Finally, $\left\Vert \cdot \right\Vert _F$ denotes the Frobenius norm operator.

## System model

For the proposed scheme in Figs. 1 and 2, the input data stream is divided into two categories, the spatial input bits (antenna pair indices) and two rotated H-8QAM symbol bits. The first input category is the spatial information. Spatial input bits ($\varvec{d}=\log _{2}N_c$) are assigned to a unique generalized $K{\rm th}$ antenna pair of transmit antennas ($k_1$ and $k_2$), which are for the *I* dimension of the system. The same $\varvec{d}$ bits are assigned to a second but different generalized $K{\rm th}$ antenna pair of transmit antennas ($k_3$ and $k_4$), which are for the *Q* dimension of the system. An example is where $N_T=4$, two possible sets of antenna pairs $$\begin{bmatrix}(1,3); (1,4); (2,3); (2,4)\end{bmatrix}$$ for the *I* dimension and $$\begin{bmatrix}(2,4); (2,3); (1,4); (1,3)\end{bmatrix}$$ for the *Q* dimension are assigned the same bit indices $$\begin{bmatrix}00; 01; 10; 11\end{bmatrix}$$. A mapping table of the grouped bits (*I* dimension transmit antenna pairs and *Q* dimension transmit antenna pair combinations), is given in Tables 1 and 2. The antenna mapping for the *I* and *Q* dimensions are different in order to avoid *ICI* or the use of the same pair of antennas to simultaneously transmit a mixed signal of the real and imaginary parts. This enhances the *EP* of the proposed system.

**Table 1:** *I* and *Q* dimension antenna combinations and mapping ($N_T = 4$ and $N_c=4$).

Spatial input bits	Antenna pair bit indices	*I*-dimension antenna pairs	*Q*-dimension antenna pairs	Rotation angle
$$\varvec{d}$$	00	$$ T_{X1},T_{X3}$$	$$ T_{X2},T_{X4}$$	0
$$\varvec{d}$$	01	$$ T_{X1},T_{X4}$$	$$ T_{X2},T_{X3}$$	$$\frac{\pi }{4}$$
$$\varvec{d}$$	10	$$ T_{X2},T_{X3}$$	$$ T_{X1},T_{X4}$$	$$\frac{\pi }{4}$$
$$\varvec{d}$$	00	$$ T_{X2},T_{X4}$$	$$ T_{X1},T_{X3}$$	0

**Table 2:** *I* and *Q* dimension antenna combinations and mapping ($N_T = 6$ and $N_c=8$).

Spatial input bits	Antenna pair bit indices	*I*-dimension antenna pairs	*Q*-dimension antenna pairs	Rotation angle
$$\varvec{d}$$	000	$$ T_{X1},T_{X2}$$	$$ T_{X5},T_{X6}$$	0
$$\varvec{d}$$	001	$$ T_{X1},T_{X3}$$	$$ T_{X4},T_{X6}$$	0
$$\varvec{d}$$	010	$$ T_{X1},T_{X4}$$	$$ T_{X3},T_{X6}$$	0
$$\varvec{d}$$	011	$$ T_{X1},T_{X5}$$	$$ T_{X2},T_{X6}$$	$$\frac{\pi }{3}$$
$$\varvec{d}$$	100	$$ T_{X1},T_{X6}$$	$$ T_{X4},T_{X5}$$	$$\frac{\pi }{3}$$
$$\varvec{d}$$	101	$$ T_{X2},T_{X3}$$	$$ T_{X1},T_{X4}$$	$$\frac{\pi }{3}$$
$$\varvec{d}$$	110	$$ T_{X2},T_{X4}$$	$$ T_{X1},T_{X3}$$	$$\frac{2\pi }{3}$$
$$\varvec{d}$$	111	$$ T_{X2},T_{X5}$$	$$ T_{X3},T_{X4}$$	$$\frac{2\pi }{3}$$

The second category of the input data stream is the symbols bit stream. A bit stream $$\varvec{b}=\begin{bmatrix} b_1 b_2 ... b_{2r}\end{bmatrix}$$, with $$r=$$log$$_2$$ *M* is fed into a mapper ($$\mathfrak {G_1}$$). In mapper ($$\mathfrak {G_1}$$), the 2*r* input bits are mapped onto a rotated H-8QAM signal constellation set to yield two symbols $$ S _{q}^{1}$$ and $$ S _{q}^{2}$$, where $$q=1+\sum _{v=1}^{2r}2^{2r-v}b_v$$, $$q\in [1:M^2]$$. The creation of the H-8QAM symbol constellation and its rotation is well discussed by Cogen et al.^17^. This paper adopts the same way of creating H-8QAM systems. Also, Cogen et al.^17^, discusses the degrading effect of error floor on the *EP* of QSM systems when using H-QAM without rotation. Thus, Cogen et al.^17^, then devised a solution of using optimum rotation angles on H-QAM-QSM systems to curb the error floor on the *EP* of H-QAM-QSM schemes. The optimum rotation angle for the chosen H-8QAM schemes in this paper, is $$\frac{\pi }{4}$$^17^. The mapping of the rotated H-8QAM symbols to bits is provided in Table 3 and Fig. 10. The *SE* of the proposed GQSM-H-8QAM scheme is given as,

1 $$\begin{aligned} \mathfrak {m}=2\log _{2}(M) + \lfloor \log _{2}\begin{pmatrix} N_T \\ 2\end{pmatrix} \rfloor _{2^p}. \end{aligned}$$

**Figure 3:**
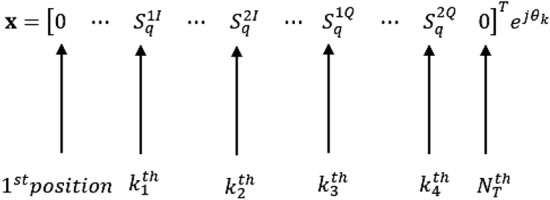
Transmitted signal vector $\textbf{x}$, for the GQSM-H-8QAM scheme.

The modulated symbols ($$ S _{q}^{1}$$ and $$ S _{q}^{2}$$) are split into their respective real parts and imaginary parts ($$ S _{q}^{1I}+ \Im { S _{q}^{1Q}}$$ and $$ S _{q}^{2I}+\Im { S _{q}^{2Q}}$$). The real parts of the symbols are transmitted via the *I* dimension using the in-phase transmit antenna pairs ($$k_1$$ and $$k_2$$). The imaginary parts of the symbols are in turn transmitted through the *Q* dimension using the quadrature antenna pairs ($$k_3$$ and $$k_4$$). Thus, the split symbols are transmitted by four transmit antennas concurrently per time slot, in the form of an $$N_T\times 1$$ transmit vector $$\textbf{x}$$ shown in Fig. 3. Vector $$\textbf{x}$$ has four non-zero elements, which are the two real parts ($$ S _{q}^{1I}$$ and $$ S _{q}^{2I}$$) and the two imaginary parts ($$ S _{q}^{1Q}$$ and $$ S _{q}^{2Q}$$) of the symbols. Moreover, the positions of these four nonzero elements correspond to the indices of the four chosen active transmit antennas. $$\theta _k$$ is the rotation angle applied to different transmit antenna pairs as shown in Tables 1 and 2. This is done in order to further minimise *ICI* that could be generated by overlapping antenna pairs^14^. The discussion of the selection of the antenna pairs and the optimum $$\theta _k$$ was discussed in^14^ and^19^. Hence, Tables 1 and 2 show the chosen transmit antenna pairs and the corresponding optimum $$\theta _k$$ values used in this paper according to^14^.

**Table 3:** rotated H-8QAM symbol mapping table.

Data symbol input bits	*q*	Rotated H-8QAM symbol ($S$)
000	0	$-1+1i$
001	1	$+1+3i$
010	2	$+1-1i$
011	3	$+3+1i$
100	4	$-3+3i$
101	5	$-3-1i$
110	6	$-1-3i$
111	7	$+3-3i$

At the receiver, the signal is given by $\textbf{y}\in \mathbb {C}^{N_R\times 1}$ (2), as

2 $$\begin{aligned} \textbf{y}&=\sqrt{\frac{\rho }{4}}\textbf{H}\textbf{x} + \textbf{n}, \nonumber \\&=\sqrt{\frac{\rho }{4}}\bigg (\left( \textbf{h}_{k_1} S _{q}^{1I} + \textbf{h}_{k_2} S _{q}^{2I}\bigg )e^{j\theta _k} + \Im \bigg (\textbf{h}_{k_3} S _{q}^{1Q} + \textbf{h}_{k_4} S _{q}^{2Q}\bigg )e^{j\theta _k} \right) + \textbf{n}, \end{aligned}$$

where the fading channel matrix is represented by $\textbf{H}\in \mathbb {C}^{N_R\times N_T}$ and $\textbf{n}\in \mathbb {C}^{N_R\times 1}$ is the AWGN noise vector. $\textbf{h}_{k_l}(1\le l\le N_T)$ is the *l*th column vector of the channel gain matrix $$\textbf{H}=\begin{bmatrix}\textbf{h}_{k_1}&\textbf{h}_{k_2}&\textbf{h}_{k_3}&...&\textbf{h}_{k_{N_T}}\end{bmatrix}$$ and $$\textbf{h}_{k_l}=\begin{bmatrix}\textbf{h}_{1k_l}&\textbf{h}_{2k_l}&\textbf{h}_{3k_l}&...&\textbf{h}_{N_Rk_l}\end{bmatrix}^T$$. The elements of both $\textbf{n}$ and $\textbf{H}$ are assumed to be i.i.d Gaussian random variables (*GRVs*) with distribution $CN (0,1)$, respectively. Finally, $\frac{\rho }{4}$ is the average signal-to-noise ratio (*SNR*) per receive antenna.

**Figure 4:**
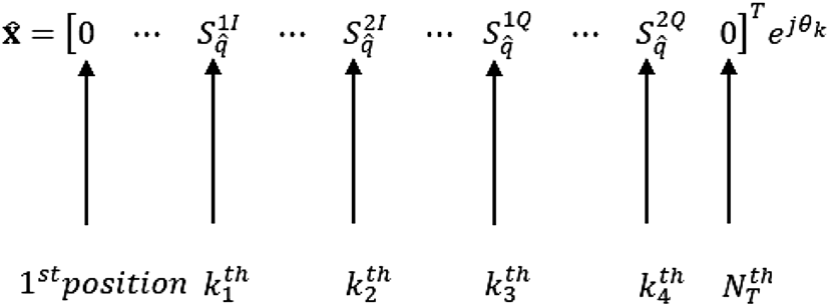
Estimated received signal vector for the GQSM-H-8QAM scheme.

The optimum maximum likelihood detection (*MLD*) for the proposed scheme, can be written as in (3) and it entails a joint estimation of the transmission antenna pairs indices ($\hat{K}$) and the sent symbols ($S _{\hat{q}}^{1}$ and $S _{\hat{q}}^{2}$)^11^ and^12^, as follows,

3 $$\begin{aligned} \begin{bmatrix} \hat{K}, S _{\hat{q}}^{1}, S _{\hat{q}}^{2}\end{bmatrix}&=\underset{K\in 1:N_c}{\underset{q\in 1:M^2}{{\text {argmin}}}}\bigg \{{\left\Vert \textbf{y}-\sqrt{\frac{\rho }{4}}\textbf{H}\hat{\textbf{x}}\right\Vert _F^2}\bigg \}, \nonumber \\&=\underset{K\in 1:N_c}{\underset{q\in 1:M^2}{{\text {argmin}}}}\bigg \{{\left\Vert \mathbf {\mathfrak {g}}\right\Vert _F^2}-2\Re \bigg \{\textbf{y}^H\mathbf {\mathfrak {g}}\bigg \}\bigg \}, \end{aligned}$$

where $\mathbf {\mathfrak {g}}=\sqrt{\frac{\rho }{4}}\bigg (\left( \textbf{h}_{k_1} S _{q}^{1I} + \textbf{h}_{k_2} S _{q}^{2I}\bigg )e^{j\theta _k} + \Im \bigg (\textbf{h}_{k_3} S _{q}^{1Q} + \textbf{h}_{k_4} S _{q}^{2Q}\bigg )e^{j\theta _k} \right)$ and $\hat{\textbf{x}}$ is the estimated received vector shown in Fig. 4, respectively.

## MLD CCA of the proposed GQSM-H-8QAM scheme-based on real valued multiplications and additions

The *CCA* of the GQSM-H-8QAM scheme is similar to that discussed in Holoubi et al.^20^. It is based on real value multiplications and additions. In^20^, it is given as $(20N_R-1)2^{\mathfrak {m}}$. In this section, all the incorporated *CCA*s of various schemes in this paper (including enhanced spectral efficiency GSM (ESE-GSM), GSM multiplexing two symbols(MIMO-GSM) and constellation reassigned GSM (GSM-CR)), have been discussed before in^6^ and^21^. As seen in Tables 4 and 5, the GQSM-H-8QAM scheme has the highest CC as compared to all the schemes included in this paper. GQSM-H-8QAM has an optimal *MLD* at the expense of a high *CC*. However, it is essential to also consider that the proposed scheme uses fewer transmit antennas as compared to various MIMO-SM schemes under the same conditions. An example is shown in Tables 9 and 10 found in "Appendix 1". Table 10 shows that the proposed scheme requires 4 transmit antennas to achieve a *SE* of $\mathfrak {m}=8$ *bits*/*s*/*Hz* as compared to C-SM and C-GSM with $N_T=32$ and 10, to achieve the same *SE* under the same conditions, respectively. Hence, the disadvantage of a high *CC* in the proposed scheme, is compensated by the advantage of using a less number of transmit antennas as compared to other various MIMO-SM schemes incorporated in this paper. Due to a high *CC* shown by the proposed scheme, as upcoming work, we intend to formulate low complexity detection algorithms to overcome this disadvantage.

**Table 4:** *MLD* *CCA* under the same conditions ($N_T=4$, $N_R=4$ and $\mathfrak {m}=8$ *bits*/*s*/*Hz*).

Scheme	*CCA* Formulae	Simulation parameters	*CC*
GQSM-H-8QAM	$(20N_R-1)2^{\mathfrak {m}}$	$M=8$	20224
GCQSM	$2^{\mathfrak {m}}(8N_R)$	$M=8$	8192
C-SM	$N_TM(3N_R+1)$	$M=64$	3328
C-QSM	$2^{\mathfrak {m}}(8N_R)$	$M=16$	8192
C-GSM	$N_RM(N_A+2)N_c$	$M=64, N_c=4, N_A=2$	4096
GSM-CR	$N_RM(N_A+2)N_c$	$M=64, N_c=4, N_A=2$	4096
MIMO-GSM	$N_RM^{N_A}(N_A+2)N_c$	$M=8, N_c=4, N_A=2$	4096

**Table 5:** *MLD* *CCA* ratios under the same conditions ($N_T=4$, $N_R=4$ and $\mathfrak {m}=8$ *bits*/*s*/*Hz*).

Ratios ($\mathfrak {r}$)	Ratios scheme	Ratio formulae	*CC* ratio
$\mathfrak {r}_1$	$\frac{GQSM-H-8QAM}{C-SM}$	$\frac{(20N_R-1)2^{\mathfrak {m}}}{N_TM(3N_R+1)}$	6.08
$\mathfrak {r}_2$	$\frac{GQSM-H-8QAM}{GCQSM}$	$\frac{(20N_R-1)2^{\mathfrak {m}}}{8N_R}$	4.94
$\mathfrak {r}_3$	$\frac{GQSM-H-8QAM}{C-GSM}$	$\frac{(20N_R-1)2^{\mathfrak {m}}}{N_cM(N_A+2)}$	4.94
$\mathfrak {r}_4$	$\frac{GQSM-H-8QAM}{GSM-CR}$	$\frac{(20N_R-1)2^{\mathfrak {m}}}{N_cM(N_A+2)}$	2.47
$\mathfrak {r}_5$	$\frac{GQSM-H-8QAM}{MIMO-GSM}$	$\frac{(20N_R-1)2^{\mathfrak {m}}}{N_cM^{N_a}(N_A+2)}$	4.94
$\mathfrak {r}_6$	$\frac{GQSM-H-8QAM}{C-QSM}$	$\frac{20N_R-1}{8N_R}$	2.47

## Proposed error performance analysis of GQSM-H-8QAM

In this section, the *ABEP* of GQSM-H-8QAM is formulated. The detection discussed in section "System model" follows the same approach as^6,11^ and^12^. Hence the *ABEP* of GQSM-H-8QAM is upper-bounded by

4 $$\begin{aligned} P_e \le \frac{1}{2^\mathfrak {m}} \sum _{q=1}^{2^\mathfrak {m}} \sum _{\hat{q}=1}^{2^\mathfrak {m}} \frac{1}{\mathfrak {m}^2}N\left( q,\hat{q}\right) P(\textbf{x}\rightarrow \hat{\textbf{x}}), \end{aligned}$$

where $N\left( q,\hat{q}\right)$ is the total number of bit errors for the associated pairwise error probability (*PEP*) event $P(\textbf{x}\rightarrow \hat{\textbf{x}})$ between the transmitted vector $\textbf{x}$ and the received vector $\hat{\textbf{x}}$. The *PEP* $P(\textbf{x}\rightarrow \hat{\textbf{x}})$ is formulated as

5 $$\begin{aligned} P(\textbf{x}\rightarrow \hat{\textbf{x}}|\textbf{H})=Q\left( \sqrt{\omega _g}\right) , \end{aligned}$$

where $\omega _g=\frac{\rho }{8}\left\Vert \left( \textbf{h}_{k_1}d_1 + \textbf{h}_{k_2}d_2+ \textbf{h}_{k_3}d_3 + \textbf{h}_{k_4}d_4\right) \right\Vert _F^2=\frac{\rho }{8}\left\Vert \textbf{H}_k\textbf{G}_k\right\Vert _F^2= \frac{\rho }{8}\left\Vert \textbf{H}_k\right\Vert _F^2\left\Vert \textbf{G}_k\right\Vert _F^2= \frac{\rho }{8}\left\Vert \textbf{H}_k\right\Vert _F^2\left( (d_1)^2+(d_2)^2+(d_3)^2+(d_4)^2\right)$, with $$\textbf{H}_k=\begin{bmatrix} \textbf{h}_{k_1}&\textbf{h}_{k_2}&\textbf{h}_{k_3}&\textbf{h}_{k_4}\end{bmatrix}$$, $$\textbf{G}_k=\begin{bmatrix}d_1&d_2&d_3&d_4\end{bmatrix}^T$$, $d_i=\left( S _q^{1I}- S _{\hat{q}}^{1I}\right) e^{j\theta _k}$ and $d_{(l+2)}=\left( S _q^{lQ}- S _{\hat{q}}^{lQ}\right) e^{j\theta _k}$, $\{i,l\}\in [1:2]$. Similarly, based on (11) in Koc et al.^22^, $\omega _g$ are chi-squared RVs with $2N_R$ degrees of freedom defined as $\omega _g=\sum _{t=1}^{2N_R} \alpha _{\omega _{g,t}}^2$ with $\alpha _{\omega _{g,t}}^2\sim N(0,\sigma _{\omega _g}^2)$ and $\sigma _{\omega _g}^2=\frac{\rho }{8}\big ((d_1)^2+(d_2)^2+(d_3)^2+(d_4)^2\big )$. The derivation of the *PEP* is found in "Appendix 2".

Upon further simplification by taking the expected value of $\omega _g$, the average *PEP* assuming $N_R$ receive antennas is adopted and modified from^6,11^ and^12^, to give

6 $$\begin{aligned} P(\textbf{x}\rightarrow \hat{\textbf{x}})= \gamma ^{N_R} \sum _{k=0}^{N_R-1} \begin{pmatrix} N_R-1+k\\ k\end{pmatrix} \begin{bmatrix} 1-\gamma \end{bmatrix}^k, \end{aligned}$$

where $$\gamma =\frac{1}{2}\left( 1-\sqrt{\frac{\sigma _{\omega _g}^2}{1+\sigma _{\omega _g}^2}}\right) $$.

Hence, taking the Taylor series of (6) and using the same approach as^6^ and^20^ at high *SNR*s, (6) leads to (7) when higher-order terms are neglected.

7 $$\begin{aligned} P(\textbf{x}\rightarrow \hat{\textbf{x}})= \frac{2^{2N_R-1}\Gamma (N_R+0.5)}{\sqrt{\pi (N_R)!}}\bigg (\sigma _{\omega _g}^2\bigg )^{-N_R}. \end{aligned}$$

The overall diversity gain ($G_c$) attained by a MIMO-SM scheme has been defined in^23^, as

8 $$\begin{aligned} -G_c=\lim _{ SNR \rightarrow \infty } \bigg \{\frac{\log \big (P_b( SNR )\big )}{\log ( SNR )}\bigg \}, \end{aligned}$$

where $P_b$ denotes overall probability of error as a function of *SNR*. Hence given $P_b(SNR)=P(\textbf{x}\rightarrow \hat{\textbf{x}})$ as given in (7), then (8) becomes

9 $$\begin{aligned} -G_c=\lim _{\rho \rightarrow \infty } \bigg \{\frac{\log \big (P(\textbf{x}\rightarrow \hat{\textbf{x}})\big )}{\log (\rho )}\bigg \}. \end{aligned}$$

Hence substituting (7) into (9), leads to (10).

10 $$\begin{aligned} -G_c=\lim _{\rho \rightarrow \infty } \bigg \{\frac{\log \big (\beta \rho ^{-N_R}\big )}{\log (\rho )}\bigg \}, \end{aligned}$$

where $$\beta =\bigg (\frac{2^{2N_R-1}\Gamma (N_R+0.5)}{\sqrt{\pi (N_R)!}}\bigg )\bigg (\frac{1}{8}\bigg )^{-N_R}\bigg ((d_1)^2+(d_2)^2+(d_3)^2+(d_4)^2\bigg )^{-N_R}$$.

Since $\lim _{\rho \rightarrow \infty } \bigg (\frac{\log (\beta )}{\log (\rho )}\bigg )=0$, therefore $G_c=N_R$. Hence the diversity gain of the proposed system is $N_R$.

## Simulation and numerical results analysis

**Figure 5:**
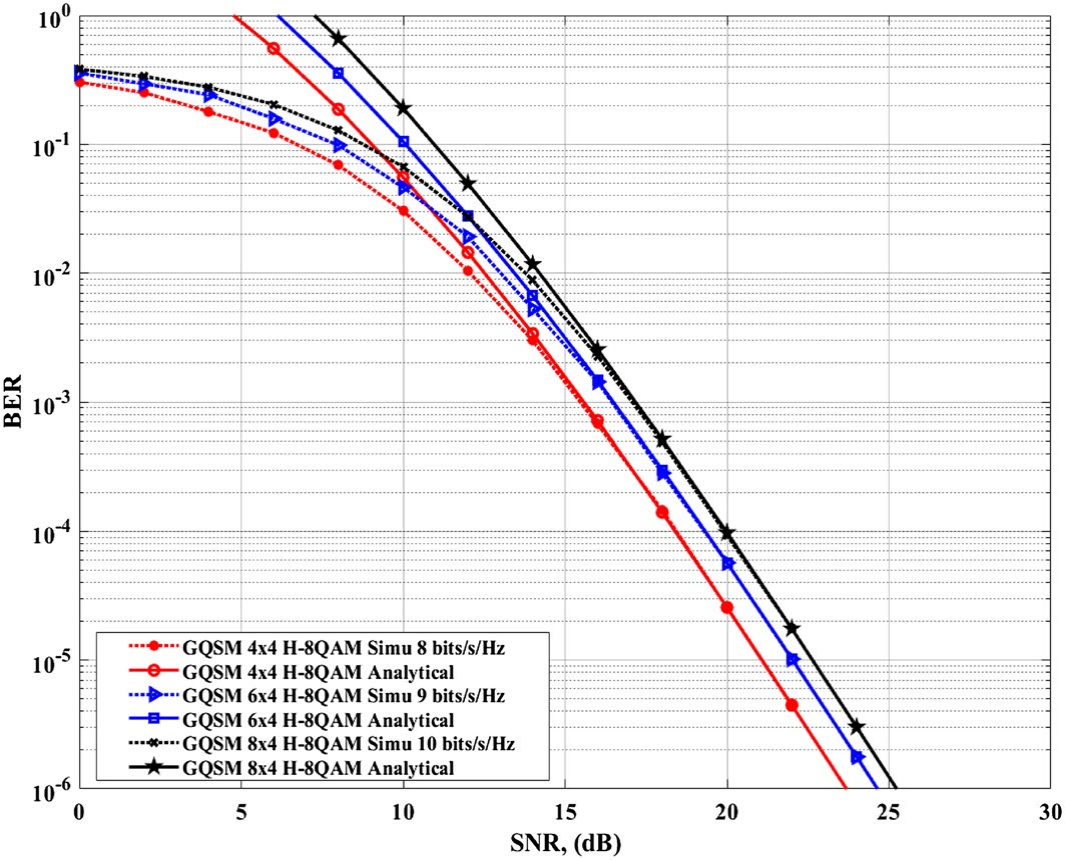
$4\times 4$, $6\times 4$ and $8\times 4$ GQSM-H-8QAM analytical and simulation ABER results.

**Figure 6:**
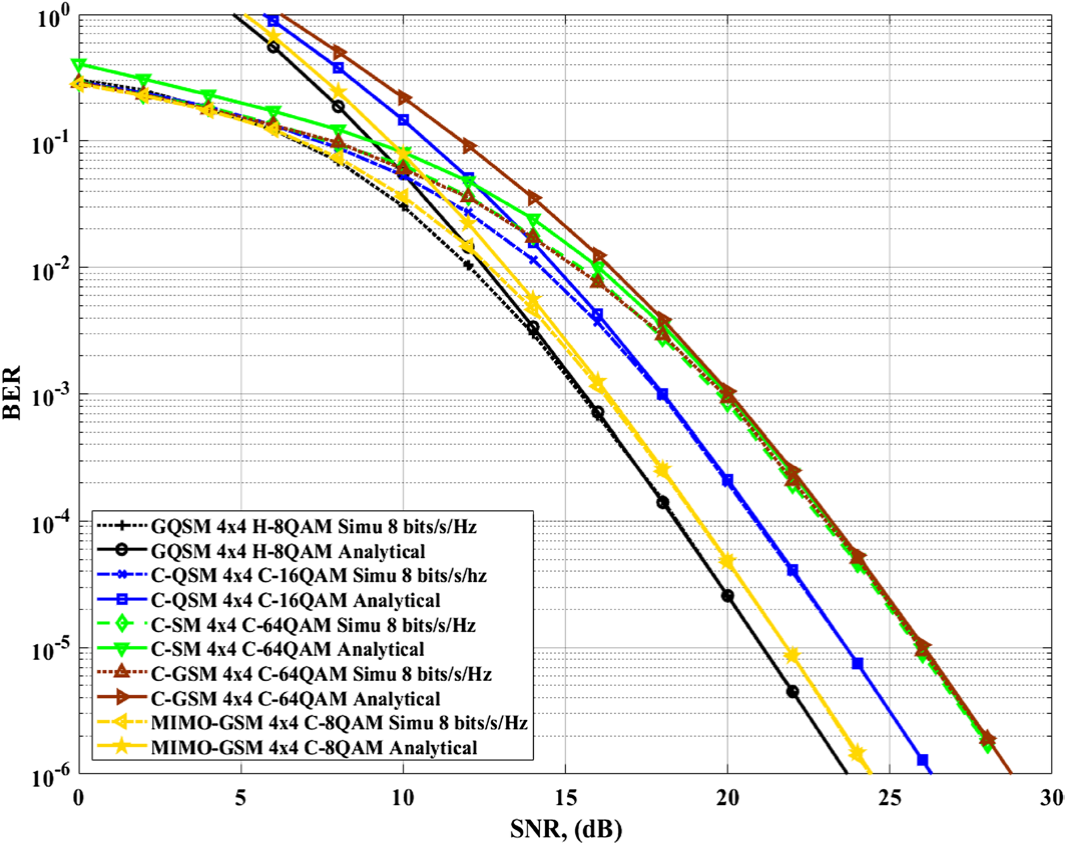
Comparison of $4\times 4$ GQSM-H-8QAM with various schemes of the same *SE*, same $N_T$ and $N_R$.

**Figure 7:**
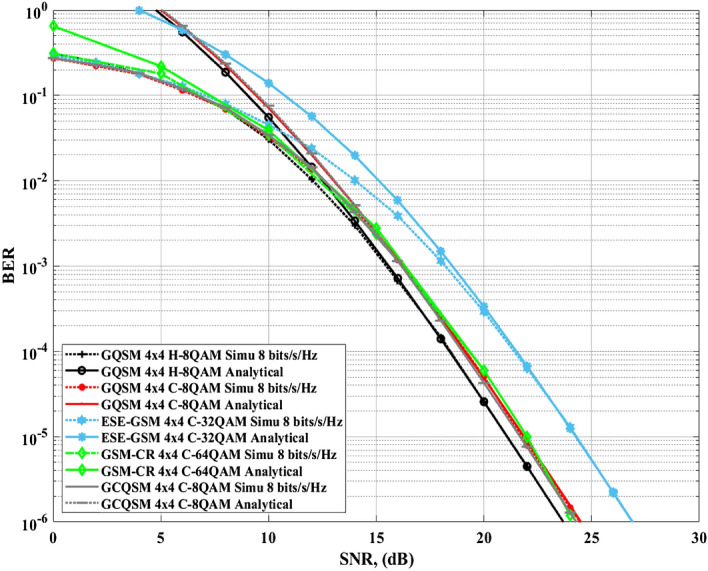
Comparison of $4\times 4$ GQSM-H-8QAM with various schemes of the same *SE*, same $N_T$ and $N_R$.

This section presents simulation results for the proposed GQSM-H-8QAM system with a different number of transmit antennas and comparisons with C-SM, C-GSM, C-QSM, MIMO-GSM and ESE-GSM. The theoretical *ABEP* for C-SM schemes was based on (9) in Naidoo et al.^4^, that of C-GSM schemes was based on (5) in Pillay et al.^19^, and that of ESE-GSM was based on (5) and (12) in Pillay et al.^19^. The theoretical *ABEP* of C-QSM was based on (3) and (4) in Oladoyinbo et al.^8^, and that of MIMO-GSM was based on (16) in Wang et al.^21^. Another aim of this section is to validate the theoretical performance bound derived in (4). The average bit error rate *BER* of the proposed scheme was evaluated using Monte Carlo simulations over i.i.d *RF-F* fading channels with AWGN. The *BER* performance was assessed for various *SE*s ($\mathfrak {m}=8$ *bits*/*s*/*Hz*, $\mathfrak {m}=9$ *bits*/*s*/*Hz* and $\mathfrak {m}=10$ *bits*/*s*/*Hz*) as a function of the average *SNR* per receive antenna ($\rho /4$). These three *SE*s considered, are shown in Figs. 5, 6, 7, 9. The comparisons were made under the same conditions, which are; four receive antennas assumed in all scenarios, identical *SE*, optimal *MLD* method, and at a *BER* value of $1\times 10^{-5}$ for comparison. The formulae for evaluating the *SE*s of all the various scheme in Figs. 5, 6, 7, 9 are found in "Appendix 1", Table 8.

Firstly, Fig. 5 presents the *BER* performance curves of the GQSM-H-8QAM scheme with $N_T=4, 6$ and 8, respectively. In Fig. 5, the analytical results are validated by simulation results and it is seen that they are tighter as the *SNR* values increase. They are tight at high SNR values because of the upper-bounded *ABER* performance in all the configurations of the GQSM-H-8QAM scheme.

Secondly, Figs. 6 and 7 with Table 6 show the simulation results and the calculated *EP* gains of the proposed $4\times 4$ GQSM-H-8QAM scheme over $4\times 4$ various schemes of the same *SE* ($\mathfrak {m}=8$ *bits*/*s*/*Hz*). As seen from Figs. 6 and 7 together with Table 6, the proposed scheme has an *EP* of 0.61 dB over $4\times 4$ GCQSM-C-8QAM, 0.93 dB over $4\times 4$ GSM-CR-64QAM scheme and 0.75 dB over $4\times 4$ MIMO-GSM-C-8QAM scheme. Also, Wang et al.^21^, discussed MIMO-GSM and it was shown to outperform C-SM and C-GSM schemes of the same *SE*. In^14^, it is also seen that GSM-CR outperforms C-SM and C-GSM schemes. Thus, the proposed scheme has performance gain over all the schemes included in this paper, as it outperformed the best of those discussed in literature^14,21^. This might be because of the use of generalised antennas and H-8QAM with a *M-MED* that improved the *EP* of the proposed scheme.

**Figure 8:**
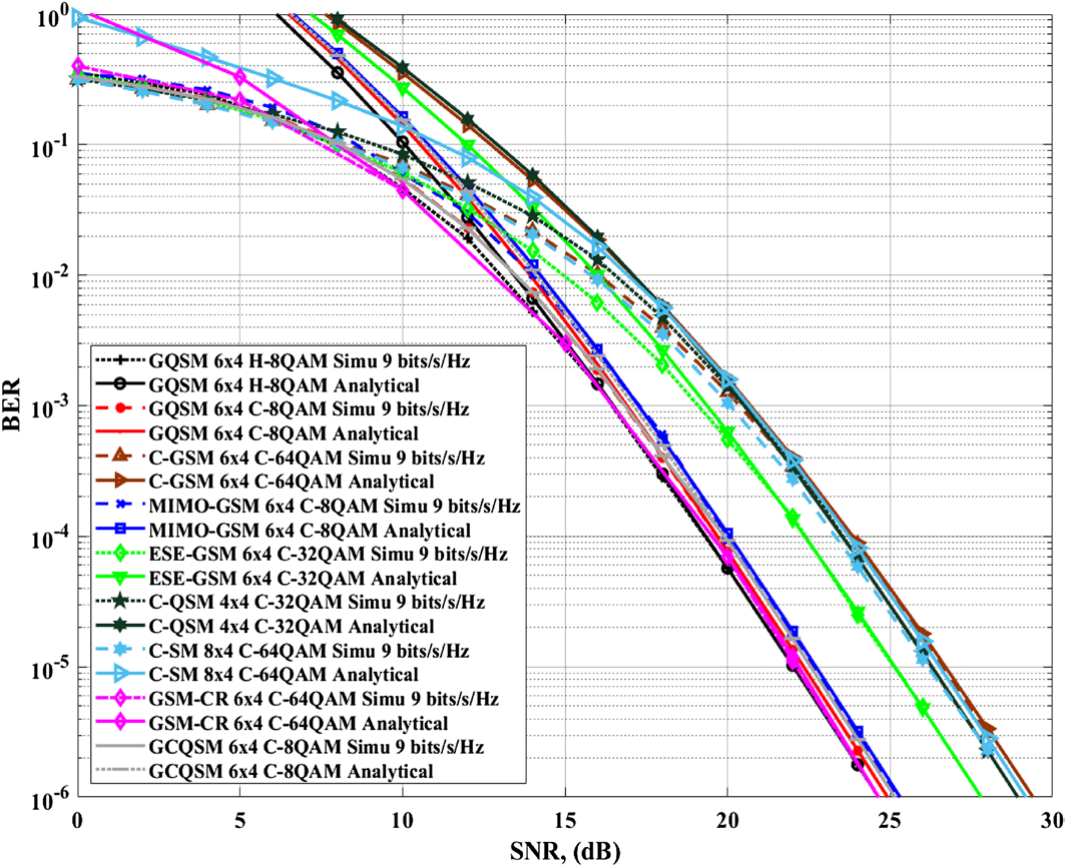
Comparison of $6\times 4$ GQSM-H-8QAM with various schemes of the same *SE*, same $N_T$ and $N_R$.

**Figure 9:**
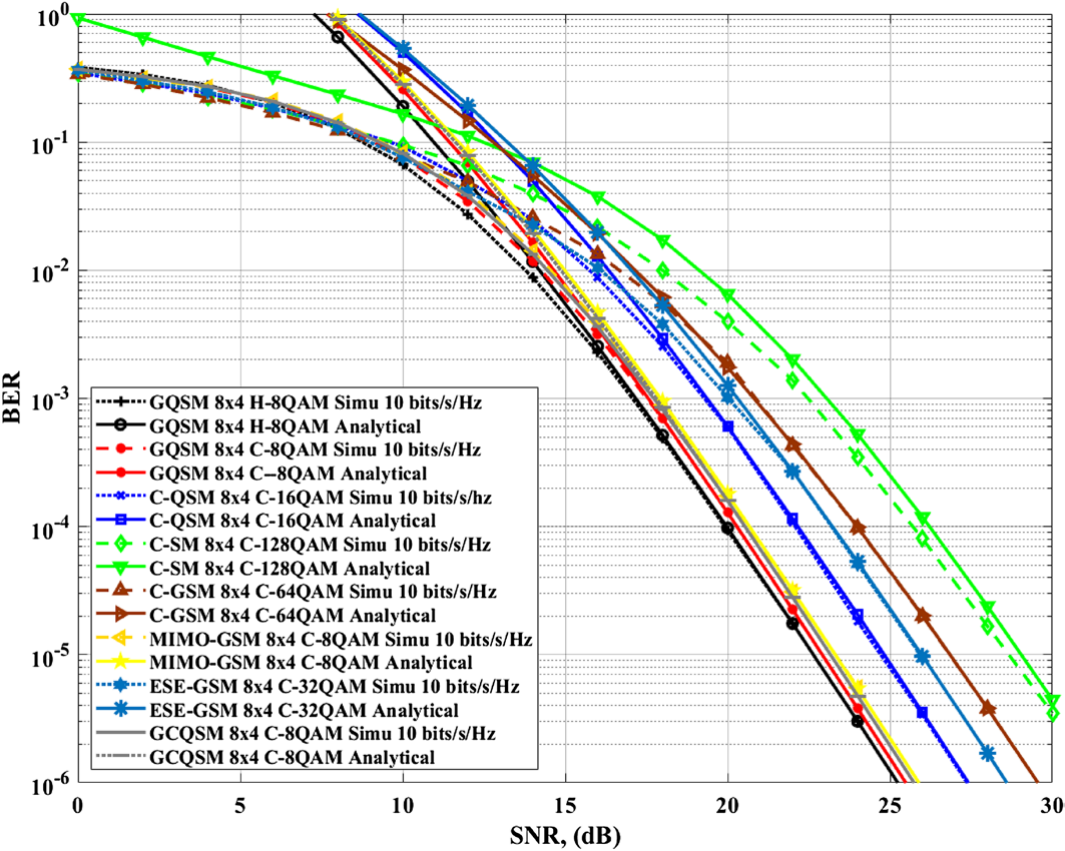
Comparison of $8\times 4$ GQSM-H-8QAM with various schemes of the same *SE*, same $N_T$ and $N_R$.

**Figure 10:**
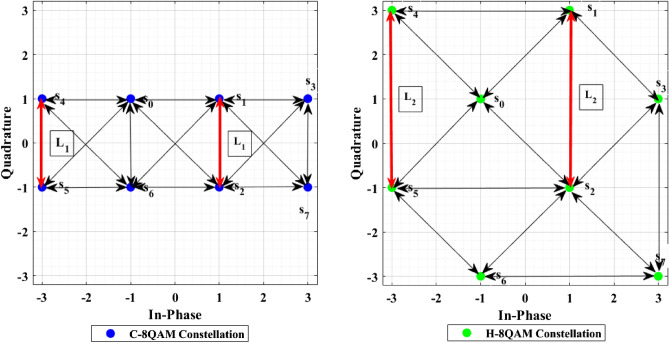
Comparison of the C-8QAM and rotated H-8QAM constellations with $L_2=\frac{4}{\sqrt{10}}>L_1=\frac{2}{\sqrt{6}}$.

Thirdly, Fig. 8 shows the proposed GQSM-H-8QAM scheme performance compared with the same schemes in Figs. 6 and 7 at a *SE* of $\mathfrak {m}=9$ *bits*/*s*/*Hz*, $N_R=4$ but under different number of transmit antennas. Fourthly, Fig. 9 shows the proposed GQSM-H-8QAM scheme performance compared with the same schemes in Fig. 6 but under different *SE*s and different number of transmit antennas. In Fig. 9, the $8\times 4$ GQSM-H-8QAM scheme is compared with other various $8\times 4$ schemes with a *SE* of $\mathfrak {m}=10$ *bits*/*s*/*Hz*. Figure 9 together with Table 7 also exhibits the same behavior as Figs. 6 and 7. The proposed scheme outperformed all the various schemes that were compared to it (Fig. 10).

It is also important, to note that the proposed scheme (GQSM-H-8QAM), has another advantage of using a less number of transmit antennas under conditions of fixed $N_R$ and fixed *M* at a certain *SE* ($\mathfrak {m}$) as compared to the other systems included in this paper. An example is shown in Tables 9 and 10 found in "Appendix 1", which show the number of $N_T$s required to achieve a certain *SE* at $N_R=4$. Table 9 shows that the GQSM-H-8QAM scheme requires 4 transmit antennas to achieve a *SE* of $\mathfrak {m}=8$ *bits*/*s*/*Hz* using H-8QAM as compared to C-SM which requires $N_T=32$, C-GSM which requires $N_T=10$ and ESE-GSM which requires $N_T=8$ to achieve the same *SE* under the same settings. This shows that the GQSM-H-8QAM scheme has an advantage of less hardware costs as it requires a less number of transmit antennas as compared to the other systems. The only exception seen in Tables 9 and 10, is the MIMO-GSM scheme which requires the same number of transmit antennas as the proposed scheme to achieve the same *SE* under identical settings as the proposed scheme. However, it is outperformed by the proposed scheme as seen from the results in Figs. 6, 7, 9.

**Table 6:** $4\times 4$ GQSM-H-8QAM *EP* gains over $4\times 4$ various schemes of the same $\mathfrak {m}=8$ bits/s/Hz.

Scheme	GQSM-H-8QAM proposed scheme (dB) gain
$4\times 4$ GQSM-C-8QAM	0.68
$4\times 4$ GCQSM-C-8QAM	0.61
$4\times 4$ C-QSM-C-16QAM	2.58
$4\times 4$ C-SM-C-64QAM	4.78
$4\times 4$ C-GSM-C-64QAM	4.85
$4\times 4$ GSM-CR-C-64QAM	0.93
$4\times 4$ MIMO-GSM-C-8QAM	0.75
$4\times 4$ ESE-GSM-C-32QAM	3.22

**Table 7:** $8\times 4$ GQSM-H-8QAM *EP* gains over various $8\times 4$ schemes of the same $\mathfrak {m}=10$ *bits*/*s*/*Hz*.

Scheme	GQSM-H-8QAM proposed scheme (dB) gain
$8\times 4$ GQSM-C-8QAM	0.27
$8\times 4$ GCQSM-C-8QAM	0.43
$8\times 4$ C-QSM-C-16QAM	2.08
$8\times 4$ C-SM-C-64QAM	6.02
$8\times 4$ C-GSM-C-64QAM	4.19
$8\times 4$ MIMO-GSM-C-8QAM	0.68
$8\times 4$ ESE-GSM-C-32QAM	3.29

## Conclusion

In this paper, GQSM-H-8QAM scheme was proposed to improve the *EP* of MIMO-SM schemes. The scheme builds on GCQSM and uses energy efficient H-8QAM instead of the C-8QAM, to improve the *EP* of MIMO-SM schemes. Also, in this paper, an upper bound analytical *EP* formula for the proposed scheme in i.i.d (*RF-F*) fading channels was derived. This analytical bound was validated using Monte Carlo simulations. The results showed that the derived analytical bound is increasingly tight with increasing *SNR* values. Furthermore, the proposed scheme outperformed GCQSM, C-SM, C-QSM, MIMO-GSM, C-GSM and other various MIMO-SM schemes incorporated in this paper. Hence, from the results obtained, it can be concluded that the proposed scheme is a viable option of improving the *EP* of MIMO-SM schemes. However, despite its capability of improving the *EP* of MIMO-SM schemes, the proposed scheme was found to have a high complexity *MLD*. Hence, for future work the authors intend to work on low complexity detection methods for the proposed scheme. In addition, the authors also intend to build on the proposed scheme by fusing it with uncoded space-time labelling diversity and golden code-words to improve/enhance the *SE* of MIMO-SM systems.

## Data Availability

All data generated or analysed during this study is included in this published article [and its supplementary information files].

## Appendices

### H-8QAM constellation set

See Fig. 10, Table 8.

**Table 8:** List of formulae for calculating the *SE* of various schemes^14,19^ and^21^.

Model scheme	Formula
GQSM-H-8QAM	$$\mathfrak {m}=2\log _{2}(M) + \lfloor \log _{2}\begin{pmatrix} N_T \\ N_A\end{pmatrix} \rfloor _{2^p}$$
GCQSM	$$\mathfrak {m}=\log _{2}(M) + \lfloor \log _{2}\begin{pmatrix} N_T \\ 2N_A\end{pmatrix} \rfloor _{2^p}$$
C-SM	$\mathfrak {m}=\log _{2}(M) + \log _{2}(N_T)$
C-GSM	$$\mathfrak {m}=\log _{2}(M) + \lfloor \log _{2}\begin{pmatrix} N_T \\ N_A\end{pmatrix} \rfloor _{2^p}$$
GSM-CR	$$\mathfrak {m}=\log _{2}(M) + \lfloor \log _{2}\begin{pmatrix} N_T \\ N_A\end{pmatrix} \rfloor _{2^p}$$
MIMO-GSM	$$\mathfrak {m}=2\log _{2}(M) + \lfloor \log _{2}\begin{pmatrix} N_T \\ N_A\end{pmatrix} \rfloor _{2^p}$$
C-QSM	$\mathfrak {m}=\log _{2}(M) + 2\log _{2}(N_T)$
ESE-GSM	$$\mathfrak {m}=\log _{2}(M) + 1 + \lfloor \log _{2}\begin{pmatrix} N_T \\ N_A\end{pmatrix} \rfloor _{2^p}$$

For the following Tables 9 and 10, where other schemes are excluded, means that they were not compatible for the conditions under comparison.

**Table 9:** Number of $N_T$ required for $\mathfrak {m}=8$ *bits*/*s*/*Hz* for $M=8$ and $N_R$ $=4$.

Model scheme	GQSM-H-8QAM	C-SM	C-GSM	GSM-CR	MIMO-GSM	ESE-GSM
$N_T$	4	32	10	10	4	8

**Table 10:** Number of $N_T$ required for $\mathfrak {m}=10$ *bits*/*s*/*Hz* for $M=8$ and $N_R$ $=4$.

Model scheme	GQSM-H-8QAM	C-SM	C-GSM	GSM-CR	MIMO-GSM	ESE-GSM
$N_T$	8	128	18	18	8	12

### Derivation of the *PEP*

Based on the equivalent system model in (3), the conditional PEP, $P(\textbf{x}\rightarrow \hat{\textbf{x}})$ can be formulated as

A.11 $$\begin{aligned} P(\textbf{x}\rightarrow \hat{\textbf{x}}|\textbf{H})=P\left( \left\Vert \textbf{y}-\sqrt{\frac{\rho }{4}}\textbf{H}\varvec{x}\right\Vert _F^2>\left\Vert \textbf{y}-\sqrt{\frac{\rho }{4}}\textbf{H}\hat{\textbf{x}}\right\Vert _F^2\right) . \end{aligned}$$

Substituting (2) into (A.11) leads to

A.12 $$\begin{aligned} P(\textbf{x}\rightarrow \hat{\textbf{x}}|\textbf{H})=P\left( \textbf{A}>\textbf{B}\right) , \end{aligned}$$

where

$$\begin{aligned} \textbf{A}= & {} \left\Vert \textbf{n}\right\Vert _F^2,\\ \textbf{B}= & {} \frac{\rho }{4}\left\Vert \textbf{h}_{k_1}d_1 + \textbf{h}_{k_2}d_2+ \textbf{h}_{k_3}d_3 + \textbf{h}_{k_4}d_4+\textbf{n}\right\Vert _F^2, \end{aligned}$$

$$d_i=\left( S _q^{1I}- S _{\hat{q}}^{1I}\right) e^{j\theta _k}$$ and $$d_{(l+2)}=\left( S _q^{lQ}- S _{\hat{q}}^{lQ}\right) e^{j\theta _k}$$, $$\{i,l\}\in [1:2]$$.

Equation (A.12) can also be rewritten as;

A.13 $$\begin{aligned} P(\textbf{x}\rightarrow \hat{\textbf{x}}|\textbf{H})=P\left( \textbf{A}>\textbf{C}+\textbf{A}+\textbf{D}\right) , \end{aligned}$$

where

$$\begin{aligned} \textbf{C}=\frac{\rho }{4}\left\Vert \textbf{h}_{k_1}d_1 + \textbf{h}_{k_2}d_2+ \textbf{h}_{k_3}d_3 + \textbf{h}_{k_4}d_4\right\Vert _F^2, \end{aligned}$$

and

$$\begin{aligned} \textbf{D}=2Re\{\sqrt{\frac{\rho }{4}}\textbf{n}^H\left( \textbf{h}_{k_1}d_1 + \textbf{h}_{k_2}d_2+ \textbf{h}_{k_3}d_3 + \textbf{h}_{k_4}d_4\right) \}. \end{aligned}$$

Simplifying Equation (A.13) leads to (A.14).

A.14 $$\begin{aligned} P(\varvec{x}\rightarrow \hat{\textbf{x}}|\textbf{H})=P\left( \textbf{D}<\textbf{C}\right) . \end{aligned}$$

Since $\textbf{n}^H\in \mathbb {C}^{1\times N_R}$ and each entry of $$\textbf{n}^H=\begin{bmatrix} n_1^*&n_2^*&n_3^*&...&n_{N_R}^*\end{bmatrix}$$ is Gaussian distributed, it therefore means that each entry of $\Re \bigg \{\sqrt{\frac{\rho }{4}}\textbf{n}^H\textbf{h}_{k_z}d_z\bigg \}$, $z\in [1:4]$ is also a *GRV* with distribution $N\left( 0,\frac{\rho }{4}\left| d_z\right| ^2\sum _{l=1}^{N_R}\left| h_{k_z}^l\right| ^2\right)$. Hence, this implies that our decision variable is also Gaussian distributed as $\Re \bigg \{\sqrt{\frac{\rho }{4}}\textbf{n}^H\bigg (\textbf{h}_{k_1}d_1 + \textbf{h}_{k_2}d_2+ \textbf{h}_{k_3}d_3 + \textbf{h}_{k_4}d_4\bigg )\bigg \}\sim N\left( 0,\frac{\rho }{8}\left\Vert \textbf{h}_{k_1}d_1 + \textbf{h}_{k_2}d_2+ \textbf{h}_{k_3}d_3 + \textbf{h}_{k_4}d_4\right\Vert _F^2\right)$ and it leads to

A.15 $$\begin{aligned} P(\textbf{x}\rightarrow \hat{\textbf{x}}|\textbf{H})=Q\left( \frac{\omega _g}{\sqrt{\omega _g}}\right) =Q\left( \sqrt{\omega _g}\right) , \end{aligned}$$

where $\omega _g=\frac{1}{2}\textbf{C}=\frac{\rho }{8}\left\Vert \textbf{H}_k\textbf{G}_k\right\Vert _F^2=\frac{\rho }{8}\left\Vert \textbf{H}_k\right\Vert _F^2\left\Vert \textbf{G}_k\right\Vert _F^2=\frac{\rho }{8}\left\Vert \textbf{H}_k\right\Vert _F^2\left( (d_1)^2+(d_2)^2+(d_3)^2+(d_4)^2\right)$,with $$\textbf{H}_k=\begin{bmatrix} \textbf{h}_{k_1}&\textbf{h}_{k_2}&\textbf{h}_{k_3}&\textbf{h}_{k_4}\end{bmatrix}$$ and $$\textbf{G}_k=\begin{bmatrix}d_1&d_2&d_3&d_4\end{bmatrix}^T$$.
